# Influence of astaxanthin, emulsifier and organic phase concentration on physicochemical properties of astaxanthin nanodispersions

**DOI:** 10.1186/1752-153X-7-127

**Published:** 2013-07-22

**Authors:** Navideh Anarjan, Imededdine Arbi Nehdi, Chin Ping Tan

**Affiliations:** 1 Department of Engineering, East Azarbaijan Science and Research Branch, Islamic Azad University, Tabriz, Iran; 2 College of Science, Chemistry Department, King Saud University, Riyadh 1145, Saudi Arabia; 3 Department of Food Technology, Faculty of Food Science and Technology, Universiti Putra Malaysia, UPM Serdang, Selangor 43400, Malaysia

**Keywords:** Astaxanthin, Emulsification-evaporation, Nanodispersion, Emulsion component

## Abstract

**Background:**

The emulsification-evaporation method was used to prepare astaxanthin nanodispersions using a three-component emulsifier system composed of Tween 20, sodium caseinate and gum Arabic. Using Response-surface methodology (RSM), we studied the main and interaction effects of the major emulsion components, namely, astaxanthin concentration (0.02–0.38 wt %, x_1_), emulsifier concentration (0.2–3.8 wt %, x_2_) and organic phase (dichloromethane) concentration (2–38 wt %, x_3_) on nanodispersion characteristics. The physicochemical properties considered as response variables were: average particle size (Y_1_), PDI (Y_2_) and astaxanthin loss (Y_3_).

**Results:**

The results indicated that the response-surface models were significantly (p < 0.05) fitted for all studied response variables. The fitted polynomial regression models for the prediction of variations in the response variables showed high coefficients of determination (R^2^ > 0.930) for all responses. The overall optimum region resulted in a desirable astaxanthin nanodispersions obtained with the concentrations of 0.08 wt % astaxanthin, 2.5 wt % emulsifier and 11.5 wt % organic phase.

**Conclusion:**

No significant differences were found between the experimental and predicted values, thus certifying the adequacy of the Response-surface models developed for describing the changes in physicochemical properties as a function of main emulsion component concentrations.

## Background

Carotenoids are natural pigments synthesized by microorganisms and plants that function as light-absorbing pigments during photosynthesis and also protect cells against photosensitization. Due to their antioxidant properties, they have received great attention in the past decades. However, their unsaturated structures make them highly sensitive to heat, oxidation, and light. Furthermore, their insolubility in water and poor oil solubility at room temperature [1] cause them to have very low bioavailabilities, so only a minor fraction of the carotenoids found in raw fruits or vegetables is absorbed in the intestines [2,3]. The carotenoid astaxanthin was selected for this study due to its unique health benefits. It has a high antioxidant capacity, being a powerful quencher of singlet oxygen and a strong scavenger of oxygen free radicals. The effectiveness of astaxanthin in the prevention of different cancers has also been proven [4,5].

The incorporation of astaxanthin into nanosystems such as nanodispersions can overcome their bioavailability problems by increasing their dissolution rate and saturation solubility due to a reduced size and increased surface area [3]. With this modern method of encapsulation technology, solubility, stability, and bioavailability of carotenoids can be considerably improved. Moreover, they will be able to be incorporated into water-based food formulations effortlessly, as nutrition value enhancer, colorant, antioxidant [1,3]. The emulsification-evaporation technique is one of the most common methods used for the preparation of carotenoid nanodispersions [2-4]. The formulation parameters of nanodispersions affect their characteristics, as do processing and environmental variables. To optimize the physicochemical properties of a nanodispersion system, all these parameters should thus be optimized.

In our previous work, we optimized the processing conditions including the pressure and number of passes through high-pressure homogenizer and the evaporation temperature to obtain nanodispersions with the minimum astaxanthin particle size and polydispersity index (PDI) and maximum astaxanthin concentration [4]. In another study, we optimized the proportions of three selected components, namely, sodium caseinate, Tween 20 and gum Arabic in the emulsifier mixture to yield the best physicochemical characteristics and stability in the resulting nanodispersions. The results indicated that the mixture of 29 wt % Tween 20, 65 wt % sodium caseinate and 6 wt % gum Arabic provided the optimum nanodispersion in terms of physicochemical and stability properties [6,7].

The objective of the present study was to systematically investigate the influence of the concentrations of the three main emulsion components, astaxanthin, emulsifier and organic phase, on the particle size, PDI and astaxanthin loss of astaxanthin nanodispersions. The Authors also optimized those components, in order to obtain nanodispersions with the least astaxanthin loss during processing and the lowest particle size and PDI using response-surface methodology (RSM). In cases where multiple variables affect the outputs, RSM is an efficient procedure for investigating the relationships between the dependant (response) and the independent variables [8]. RSM has a main advantage over the single factor analysis due to its providing the assessment of the multiple variables and their interactions effects on the responses by means of a reduced number of experiments [4,8].

## Results and Discussions

### Fitting the Response-surface equations

Response-surface analysis presented empirically significant (p < 0.05) models for the estimation of variations in particle size, PDI and astaxanthin loss as a function of astaxanthin, emulsifier and organic phase concentrations. The particle size, PDI and astaxanthin loss in all the experiments are listed in Table 1. The predicted data, listed in Table 2, were calculated using the equations obtained from the regression of experimental data. All regression coefficients, corresponding R^2^ and R^2^-adjusted, individual significance F-ratio and *p*-values of the independent variables are shown in Table 2.

**Table 1:** Matrix of the central composite design (CCD), experimental and predicted data

**Run number**	^**a**^**x**_**1**_**,%**	^**a**^**x**_**2**_**,%**	^**a**^**x**_**3**_**,%**	**Particle size (nm)**		**PDI**		**Astaxanthin loss (wt %)**	
				^**b**^**Y**_**exp.**_	^**c**^**Y**_**pre.**_	^**b**^**Y**_**exp.**_	^**c**^**Y**_**pre.**_	^**b**^**Y**_**exp.**_	^**c**^**Y**_**pre.**_
1^cp^	0.20	2.0	20	97.49	93.20	0.382	0.375	12.46	17.59
2^cp^	0.20	2.0	20	95.83	93.20	0.383	0.375	15.11	17.59
3	0.30	1.0	30	103.90	103.91	0.300	0.310	25.41	24.90
4	0.10	1.0	10	90.29	93.63	0.246	0.241	36.27	35.32
5	0.10	3.0	30	139.10	137.65	0.485	0.501	4.33	0.65
6	0.30	3.0	10	107.57	112.60	0.526	0.519	26.75	25.07
7	0.10	1.0	30	99.43	94.75	0.241	0.223	38.64	38.26
8	0.30	1.0	10	133.20	135.00	0.310	0.306	19.00	21.43
9^cp^	0.20	2.0	20	98.35	99.93	0.374	0.364	18.04	17.32
10^cp^	0.20	2.0	20	95.98	99.93	0.387	0.364	21.59	17.32
11	0.30	3.0	30	137.88	134.89	0.549	0.566	10.50	12.18
12	0.10	3.0	10	109.73	110.07	0.393	0.432	10.98	12.23
13	0.38	2.0	20	130.97	129.00	0.443	0.445	17.99	18.57
14^cp^	0.20	2.0	20	96.98	97.35	0.372	0.376	19.81	19.04
15	0.20	2.0	38	120.02	125.26	0.388	0.399	15.65	14.80
16	0.20	2.0	2	121.97	116.30	0.341	0.353	26.76	23.29
17	0.20	3.8	20	129.96	129.62	0.672	0.644	13.69	15.16
18	0.02	2.0	20	104.83	106.37	0.315	0.308	15.34	19.52
19^cp^	0.20	2.0	20	96.41	97.35	0.384	0.376	19.53	19.04
20	0.20	0.2	20	99.14	99.05	0.209	0.223	47.56	46.91

^cp^ center point.

^a^x_1_, x_2_ and x_3_ are astaxanthin concentration, emulsifier concentration and organic phase concentration, respectively.

^b^Y_exp._ experimental data.

^c^Y_pre._ predicted data.

**Table 2:** **Regression coefficients, significance probability ( *p *-value and F-ratio**), ***R***^***2 ***^**and R**^**2**^ (**adj**) **values for the final reduced second**-**order polynomial models**

**Term **^**a**^	**Particle size (nm)**			**PDI**			**Astaxanthin loss (%)**		
	**Coefficient**	**F-value**	***p-*value**	**Coefficient**	**F-ratio**	***p-*value**	**Coefficient**	**F-ratio**	***p*-value**
Constant	133.089	82.99	0.00	0.41996	23.09	0.000	66.040	73.46	0.000
Linear									
x_1_	32.485	0.23	0.642 ^b^	0.379193	55.52	0.000	−133.685	28.91	0.000
x_2_	−19.755	8.61	0.019	0.014151	0.33	0.572^b^	−28.809	39.43	0.000
x_3_	−3.702	30.25	0.001	−0.00196	1.83	0.197^b^	0.556	5.00	0.047
Quadratic									
x_1_^2^	627.664	30.24	0.001	NS^b^	NS^b^	NS^b^	NS^b^	NS^b^	NS^b^
x_2_^2^	5.243	21.10	0.002	0.01759	15.59	0.001	5.139	3.70	0.001
x_3_^2^	0.072	40.14	0.000	NS^b^	NS^b^	NS^b^	NS	NS	NS
Interaction									
x_1_x_2_	−63.450	14.07	0.006	NS^b^	NS^b^	NS^b^	65.529	30.51	0.000
x_1_x_3_	−4.689	7.68	0.024	NS^b^	NS^b^	NS^b^	NS^b^	NS^b^	NS^b^
x_2_x_3_	0.998	34.82	0.000	0.00162	5.63	0.033	−0.39591	11.55	0.006
R^2^	0.963			0.980			0.942		
R^2^ (adj)	0.913			0.968			0.900		

^a^ x_1_, x_2_ and x_3_ are astaxanthin concentration, emulsifier concentration and organic phase concentration, respectively.

^b^ (NS) not significant (*p* > 0.05).

The high coefficients of determination (*R*^2^ > 0.90) (Table 2) obtained from the ANOVA analysis show that more than 90% of the variability of the studied physicochemical characteristics of the prepared nanodispersions was explained by the RSM models as a nonlinear function of emulsion composition concentrations.

As shown in Table 2, the emulsifier concentration had the most significant (*p* < 0.05) effect on all responses compared with other emulsion components. Although the interaction effects of astaxanthin concentration with organic phase concentration and the quadratic effect of astaxanthin were non-significant in the variation of both PDI and astaxanthin loss, the variation of particle size was influenced significantly (p < 0.05) by all quadratic and interaction effects of the variables. All nonsignificant interactions and quadratic terms were omitted in the final reduced regression models. The polynomial regression models and recommended optimum region were significant (*p* < 0.05) only in the studied independent variable ranges. Thus, the fitted models cannot be extrapolated beyond these ranges [9].

### Average particle size

**Figure 1:**
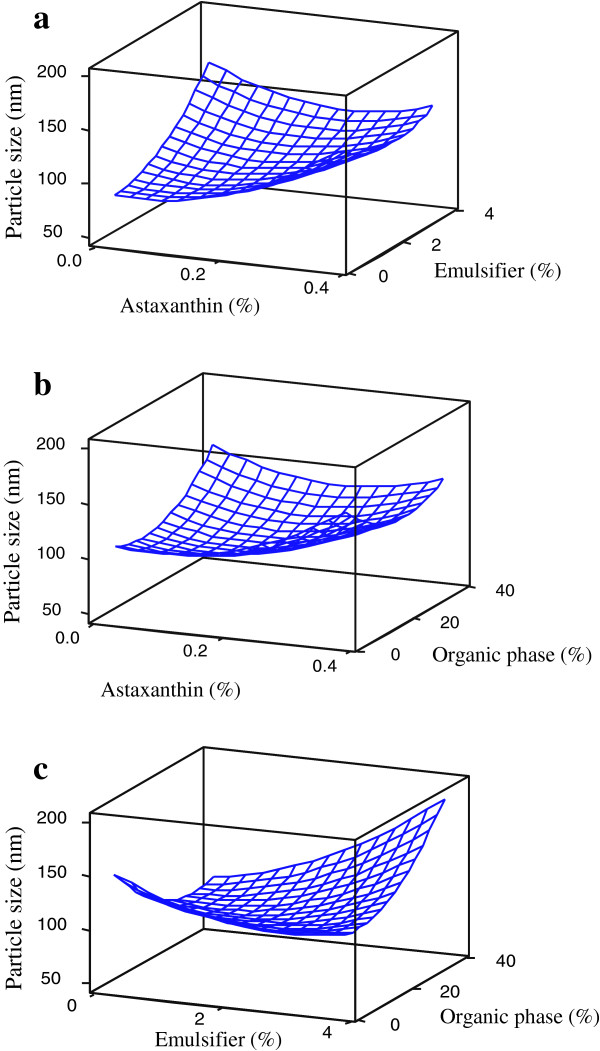
Response-surface plots for particle size as function of significant (p < 0.05) interaction effects between emulsion component concentrations.

To visualize the effect of the independent variables on responses (only for significant interactions), surface response plots of the quadratic polynomial models were generated by holding an independent variable constant at its center point and varying the remaining two variables within the experimental range. As shown in Table 2, the variation of average particle size was significantly (p < 0.05) explained by a full quadratic regression equation (R^2^ = 0.963). The main effect of astaxanthin concentration was insignificant on particle size changes, but its quadratic effect was significant on particle size. The positive quadratic effect of astaxanthin concentration confirmed the increases of particle size upon increasing the astaxanthin concentration, especially at high levels. The negative single effects and positive quadratic effects of emulsifier and organic phase concentrations on the particle size of the astaxanthin nanodispersions illustrated that the effect of this variable on particle size was different at its various levels. As shown in Figure 1 and Table 2, at low emulsifier (or organic phase) concentrations, increasing their concentrations led a decrease in particle size, but they inversely affected this response at high levels.

Figure 1a and b show that the combined effects of astaxanthin and emulsifier concentrations as well as astaxanthin and organic phase concentration on particle size depended on the level of these two factors. For both, at low astaxanthin concentrations, increasing the emulsifier or organic phase concentration caused the particle size to increase, whereas at high astaxanthin concentrations it caused a decrease in particle size up to a point and then increased it again. According to the significance probabilities of the interaction terms of the studied independent variables (Table 2), the interaction of emulsifier concentration with organic phase concentration influenced the particle size more significantly (i.e., with a lower *p*-value and a higher F-ratio) than the other interaction terms. At low organic phase concentrations, increasing the emulsifier concentrations caused the particle size to decrease, but increasing the emulsifier concentration increased the particle size at high organic phase concentrations. Actually, at low levels of organic phase, increasing the emulsifier content could not increase the viscosity of system significantly after removing the organic phase, but at high levels of organic phase, since the system was more concentrated after organic phase removal, the viscosity increase of system due to addition of emulsifier will be more considerable. In viscose systems, the mutual disruption of the dispersed phase is hindered and larger particles would initially be produced [10-13]. The same patterns were also seen in the effect of organic phase concentration at different levels of emulsifier concentration (Figure 1c).

The increase in particle size by increasing carotenoid concentration at constant concentrations of emulsifier and organic phase has also been reported in previous studies [3,10,11]. The increase of particle size in prepared nanodispersions due to increased initial loading of bioactive compounds can be explained by the fact that as the astaxanthin content increased, the available emulsifier decreased. Therefore, their stabilizing efficiency was limited, which favoured astaxanthin particles coalescence and, thus, increase of the system mean particle size [3,10-13]. In the preparation of functional lipid nanodispersions via emulsification-evaporation, high-pressure homogenization forms water-insoluble solvent droplets containing dissolved active compounds. Evaporating the solvent causes the functional lipid compound to precipitate or crystallize, and the particles formed are bound with the emulsifier. A lower concentration of solute in the solvent ensures a sufficient amount of emulsifier is present to stabilize the small precipitated or crystallized particles after evaporation, resulting in smaller final particles in the resulting nanodispersions [3]. A considerable enhancement of particle size due to an increase of astaxanthin concentration was seen in our results, especially at high levels of astaxanthin concentration.

According to the results reported by Chu et al. [3], Mainardes and Evangelista [14] and Kanafusa et al. [15], an increase in emulsifier concentration causes a decrease in particle size in emulsion systems. Other researchers such as Jafari et al. [13], Lobo and Svereika [16] and Tcholakova et al. [17] concluded that there are two effects of emulsifier concentrations on the particle size of emulsions; a surfactant-poor regime, in which particle size decreases with increasing emulsifier concentration, and a surfactant-rich regime, in which particle size does not depend on emulsifier concentration or it increases with increasing emulsifier concentration. Both of these effects were seen in our results, except at low levels of astaxanthin and high levels of organic phase ratio, in which particle size was only increased by raising the emulsifier concentration (Figures 1a and c). Based on the stabilizing function of an emulsifier, a decrease of particle size due to an increase in emulsifier concentration was expected. In the emulsification-evaporation method, in which the emulsification and stabilization of the particles are critical factors, the amount of emulsifier plays an important role in the emulsification process and in the protection stabilization of the droplets, because it can prevent the coalescence of particles [14]. An adequate amount of emulsifier is sufficient to immediately cover the whole surface area of freshly prepared droplets after high-energy homogenization as well as the particles precipitated after solvent removal, so that preventing their recoalescence [3]. However, further increasing the emulsifier, especially at low astaxanthin or high organic phase concentrations (after evaporation of this high amount of solvent), induces particle coalescence via bridging flocculation, depletion flocculation, and other mechanisms, particularly in our studied system, in which the emulsifier contained protein, a hydrocolloid and a low-molecular-weight emulsifier, as their interactions would be extensive at high concentrations [18].

Mainardes and Evangelista [14] found that an increase in the organic phase concentration led to a slight decrease in particle size; conversely, Tan and Nakajima [2] and Chu et al. [19] reported an increase in particle size with increasing organic phase concentrations. Manipulating the viscosity of the organic phase or controlling the evaporation parameters such as duration time and the rate of solvent evaporation are some of the mechanisms by which the organic phase properties can be used to control the particle size of a dispersion system [14,19]. As illustrated in Figures 1b and c, at low levels of astaxanthin and/or high levels of emulsifier concentration, increasing the organic phase concentration caused the particle size to increase; however, at low levels of emulsifier concentration this had the inverse effect on particle size. That is, both positive and negative effects of organic phase concentration on particle size were observed at high astaxanthin concentration. The individual optimum optimization procedure predicted that the minimum average particle size (86.51 nm) would be obtained at concentrations of 0.09 wt %, 0.81 wt % and 20 wt % for astaxanthin, emulsifier and organic phase.

### Polydispersity index (PDI)

**Figure 2:**
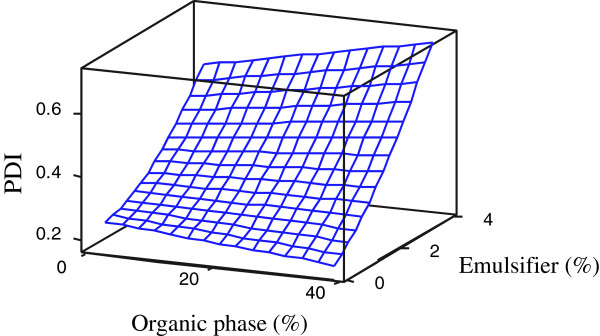
Response-surface plots for PDI as function of significant (p < 0.05) interaction effects between emulsifier and organic phase concentrations.

The results showed that the studied emulsion composition concentrations had significant (p < 0.05) effects on the PDI (Y_2_) variation (Table 2). The final reduced model showed a relatively high coefficient of determination (R^2^ = 0.978). The single effect of astaxanthin concentration, the quadratic effect of emulsifier concentration and its interaction with organic phase concentration were significant (p < 0.05) on the PDI of the resulting astaxanthin nanodispersions. The single main effects of emulsifier and organic phase concentrations were retained in final reduce model despite their insignificance due to their significant (p < 0.05) quadratic and interaction effects. All significant effects were positive, meaning that increasing these variables increased the PDI (Figure 2). By increasing the astaxanthin and/or emulsifier concentrations, the particle size distribution tended to become polymodal, perhaps due to a high recoalescence rate as a result of the higher collision of particles after emulsification or precipitation. The increase in PDI with an increasing amount of emulsifier in the system may be related to the production of micelles by the emulsifier at high concentrations. The probability of micelle production is considerably increased in multiple emulsifier systems; in these systems, the transition from a surfactant-poor regime to a surfactant-rich regime could occur at low emulsifier concentrations. Therefore, further increases in emulsifier concentration caused heterogeneity in the particle size and so increased the PDI. Several previous studies have reported different results for the effect of emulsifier concentrations on PDI; Chu et al. [3] and Mainardes and Evangelista [14] reported that the PDI was reduced by increasing the emulsifier concentration. As mentioned before, these differences in results may be related to different nature of the emulsifier systems used to stabilize the emulsion systems in the different studies. However, our finding relating to the active compound concentration effect on PDI is in good agreement with the previous results. According to our results, the organic phase concentration effect was dependent on the emulsifier concentration at different levels. At low emulsifier concentration, an increase in the organic phase concentration reduced the PDI, but at high emulsion concentrations, it acted inversely. Increasing the PDI by increasing the organic phase ratio was also reported by Chu et al. [19], but other researchers reported no systematic changes in PDI by varying the organic phase concentration [14]. The decrease of PDI by increasing the organic phase concentration (which has been observed in some of our results) may be related to the viscosity of dichloromethane, used in this work as the organic phase, being lower than the viscosity of water. A lower-viscosity organic phase is more favorable for mixing efficiency and reduces shear stress, allowing for easy droplet deformation during homogenization [18]. The individual optimum concentrations for the production of nanodispersions with the lowest PDI (0.127) were predicted to be 0.020 wt % for astaxanthin, 0.20 wt % for the emulsifier and 38% for the organic phase.

### Astaxanthin loss in nanodispersions

**Figure 3:**
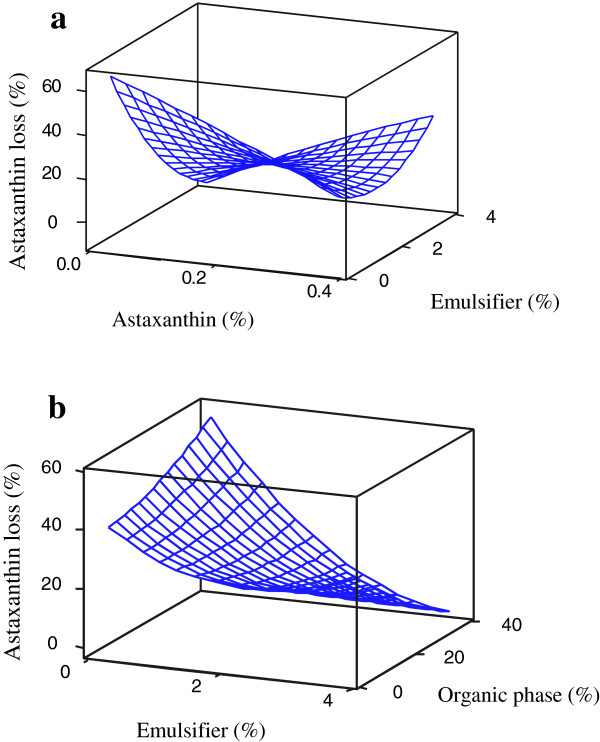
Response-surface plots for astaxanthin loss as function of significant (p < 0.05) interaction effects between emulsion component concentrations.

As shown in Table 2, the variation of astaxanthin loss (Y_3_) was significantly (p < 0.05) well-fitted by a nonlinear second-order regression equation (R^2^ = 0.942). All single main effects of the independent variables, the quadratic effect of emulsifier concentration and its interactions with both organic phase and astaxanthin concentrations were significant in the prediction of astaxanthin loss for the nanodispersions. As shown in Figure 3a, the effect of astaxanthin concentration on astaxanthin loss depended on the emulsifier concentration used. At low levels of emulsifier concentration, increasing the astaxanthin reduced astaxanthin loss, but at high concentrations of emulsifier in the system, the effect was reversed. The same pattern was observed (Figure 3a) for the influence of emulsifier concentration on astaxanthin loss at different levels of astaxanthin concentration. The effect of organic phase concentration on astaxanthin loss also was dependent on emulsifier concentration levels (Figure 3b). The effect of organic phase concentration was greater for high levels of emulsifier concentration, at which the astaxanthin loss was reduced by raising the organic phase concentration. Additionally, an extensive reduction of astaxanthin loss was observed on increasing the emulsifier concentration at high organic phase concentrations. It is well known that astaxanthin, like other carotenoids, is very sensitive to light, oxygen and heat [4,20]. The temperature rise during the emulsification-evaporation process and possible exposure to light and oxygen during processing are just some of the reasons for the loss of astaxanthin in the final prepared samples. The increasing astaxanthin loss at higher astaxanthin concentrations (at high levels of emulsifier, Figure 3a) could be the result of a relatively high probability for an isomerization or self-reaction of astaxanthin due to of the increased frequency of particle collision at high astaxanthin concentrations. Decreasing the astaxanthin loss by increasing the astaxanthin concentration (at low concentrations of emulsifier, Figure 3a) or by increasing the emulsifier and/or organic phase concentration (at high levels of organic phase and/or emulsifier concentrations, Figure 3b) may be related to the increased particle sizes in these regions. In most cases, the astaxanthin loss varied inversely with particle size; small particles possess higher surface areas for exposure to light, oxygen or free radicals than large particles and are thus more susceptible to degradation or auto-oxidation [4,21]. The reduction in astaxanthin loss with increasing emulsifier concentration (at low concentrations of astaxanthin, Figure 3a) may be because of the protective effect of the emulsifier (especially the proteins and hydrocolloids) against lipid oxidation of the active compound by altering the particle interface properties. Sodium caseinate (the main component of the emulsifier) has been reported to act as an efficient antioxidant protein by absorbing onto the droplet surface, and it exhibits a synergistic effect in combination with other antioxidant compounds [22]. The individual optimization predicted that astaxanthin nanodispersions prepared at the concentrations of 0.15 wt % astaxanthin, 3.2 wt % emulsifier and 30 wt % organic phase would produce the nanodispersions with the least astaxanthin loss (2.7 wt %).

### Optimization procedure for predicting the emulsion composition concentrations to produce the most desirable astaxanthin nanoemulsions

The astaxanthin nanodispersions with the smallest particle size and PDI and the least astaxanthin loss would be considered the optimum product. After individual numerical optimization for each response, multiple-response optimizations were also performed to simultaneously determine the optimum concentrations of emulsion components leading to the most desirable response goals. The numerical optimization showed that using 0.08 wt % astaxanthin, 2.5 wt % emulsifier and 11.5 wt % organic phase would produce an astaxanthin nanodispersion with the optimum physicochemical characteristics. Under these optimum formulation conditions, the corresponding predicted response values for average particle size, PDI and astaxanthin loss percentage were 101.76 nm, 0.354 and 13.43 wt % respectively.

### Verification of reduced model

The adequacy of the response-surface equations was checked by comparing the experimental values and the fitted values predicted by the response-regression models. The experimental and predicted values are listed in Table 1. No significant (*p* > 0.05) differences were found between these values. The agreement between the experimental and predicted values confirmed the adequacy of the corresponding response-surface models employed for describing the variation of physicochemical properties as functions of emulsion composition concentrations. For confirmation, four astaxanthin nanodispersions were also prepared according to the predicted optimum astaxanthin, emulsifier and organic phase concentrations in triplicate, and the particle size, PDI and astaxanthin loss of the prepared nanodispersions were evaluated. Tukey’s comparison test indicated insignificant differences between the experimental values and the predicted ones (Table 3) and reconfirmed the suitability of the corresponding models [10].

**Table 3:** **Experimental and predicted values of chosen points** (**obtained from optimization procedures**) **for verification of fitted reduced models**

	**Astaxanthin conc. (wt %)**	**Emulsifier conc. (wt %)**	**Organic phase conc. (%)**	**Particle size (nm)**		**PDI**		**Astaxanthin loss (%)**	
				**Y**_**exp**_	**Y**_**pre**_	**Y**_**exp**_	**Y**_**pre**_	**Y**_**exp**_	**Y**_**pre**_
1	0.09	0.081	20	90.0 ± 7.6	86.51	0.203 ± 0.009	0.215	40.07 ± 2.13	42.59
2	0.02	0.20	38	99.2 ± 3.2	97.75	0.130 ± 0.005	0.127	71.65 ± 6.02	76.14
3	0.15	3.2	30	138.7 ± 4.3	140.835	0.555 ± 0.008	0.551	3.65 ± 5.55	2.70
4	0.08	2.5	11.5	103.1 ± 5.4	101.76	0.346 ± 0.01	0.354	13.97 ± 3.96	13.43

### Stability evaluation of prepared optimum nanodispersion during storage

**Figure 4:**
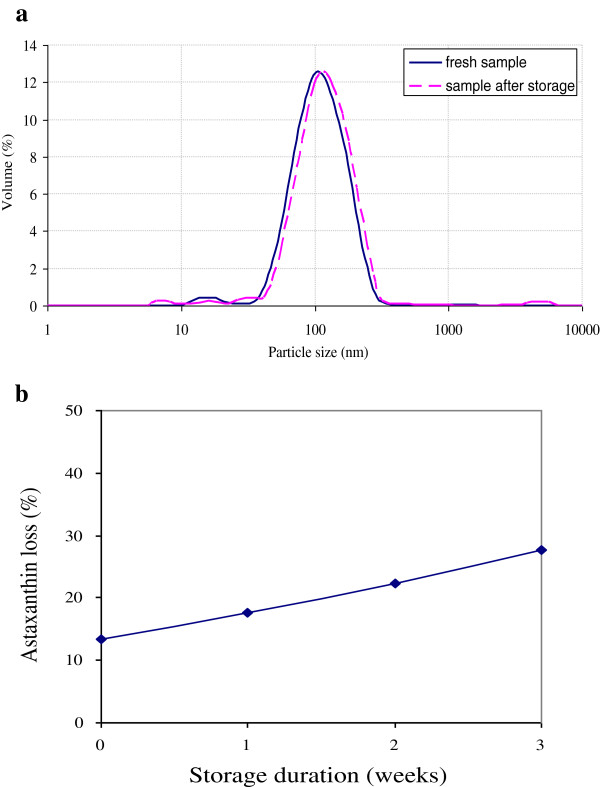
(a) Particle size distribution of obtained optimum astaxanthin nanodispersion after preparation and over 3 weeks of storage at 4°C; (b) Changes in astaxanthin content for optimum obtained astaxanthin nanodispersions during storage at 4°C.

A smaller particle size and consequently higher specific surface area of an active compound generally lead to a higher water dissolution rate and thus higher bioavailability and cellular uptake, but particles in these small size ranges (nanometers) must be stabilized physically and chemically. Changes in the mean particle size of the optimum astaxanthin nanodispersion were monitored over three weeks of storage at 4°C to evaluate their physical stability. There was no significant change in the mean particle size of the resulting optimum astaxanthin nanodispersion over three weeks (Figure 4a). We concluded that no coalescence occurred in the nanodispersion and verified the physical stability of the resulting nanodispersions. However, the significant (*p* < 0.05) decrease in astaxanthin content of the nanodispersions, either instantaneously after sample preparation or during storage, showed their limited chemical stability. Figure 4b shows the degradation profile of astaxanthin in storage at 4°C. The astaxanthin loss by the third week was 27.7%. It is well known that astaxanthin is sensitive to light, oxygen and heat, as other carotenoids [4]. Therefore, the presence of light, oxygen and heat contributed to the losses of astaxanthin during both emulsification and evaporation processes [2,4]. Furthermore, the occurrence of cavitation within the homogenizer and the production of free radicals during the nanodispersion preparation procedure along with the high surface area of the nanoparticles for exposure to this aqueous media containing free radicals are other possible causes of the degradation during sample preparation process and storage [2,23]. Addition of antioxidant compounds such as tochopherols to organic phase of nanodispersion systems and removing the oxygen or singlet oxygen generators from sample preparation and storage environment can decrease the astaxanthin degradation considerably [24].

## Conclusion

In general, this study indicated that the formulation and processing parameters had significant effects on the variation of the physicochemical properties of astaxanthin nanodispersions. The central composite design was a useful tool for optimizing the emulsion component concentrations leading to astaxanthin nanodispersions with the most desirable physicochemical properties. The high coefficients of determination obtained (R^2^ > 0.940) for the regression models by analysis of variance (ANOVA) confirmed the validity of the empirical equations developed to describe and predict the variation of the studied response variables. The results indicated that the quadratic effect of emulsifier concentration and its interaction effect with organic phase concentration were significant in the variation of all three studied responses. The optimization procedure indicated that the overall optimum emulsion component concentrations for producing the most desirable nanodispersion were 0.08 wt % astaxanthin, 2.5 wt % emulsifier and 11.5 wt % organic phase. The experimental values were shown to be in good agreement with the predicted values, thus indicating the adequacy of the fitted models. This obtained astaxanthin nanodispersion showed high physical stability and relatively acceptable chemical stability.

## Materials and methods

### Materials

Astaxanthin (> 90%) was purchased from Kailu Ever Brilliance Biotechnology Co., Ltd. (Beijing, China). Polyoxyethylene sorbitan monolaurate (Tween 20), sodium azide, sodium caseinate, analytical and HPLC-grade dichloromethane, methanol and acetonitrile were provided by Fisher Scientific (Leicestershire, UK). Gum Arabic was acquired from Merck (Darmstadt, Germany). All chemicals were used without further purification.

### Preparation of astaxanthin nanodispersions

Astaxanthin nanodispersions were prepared by the emulsification-evaporation technique. Different concentrations of an emulsifier mixture (0.2–3.8 wt %, consisting of 29 wt % Tween 20, 65 wt % sodium caseinate and 6 wt % gum Arabic) were dissolved in 0.05 M phosphate buffer (pH 7) at 40°C containing 0.02 wt % sodium azide. The emulsifiers were first dissolved in the aqueous phase under magnetic stirring for 5 h. Astaxanthin was dissolved in dichloromethane at different concentrations (0.02–0.38 wt %) to compose the organic phase, which was added to the aqueous phase at 2–38 wt %. The premix was homogenized using a conventional homogenizer (Silverson, L4R, Buckinghamshire, UK) at 5,000 rpm for 5 min. The resulting coarse emulsion then was passed twice through a high-pressure homogenizer (APV, Crawley, UK) at 50 MPa. The solvent was then removed from the fine emulsion by rotary evaporation (Eyela NE-1001, Tokya Rika Kikai Co. Ltd., Tokyo, Japan) at 25 kPa and 47°C. The applied processing conditions were determined by optimization in previous works [6,20]. The formation of astaxanthin particles took place as dichloromethane diffused into through the aqueous phase and evaporated at the water/air interface [25]. During the evaporation process, only the dichloromethane was removed from the samples. The experimental design matrix is shown in Tables 1 and 4.

**Table 4:** **Levels of independent variables established according to the central composite design** (**CCD**)

**Variable**	**Independent variable levels**				
**Independent variables**	**Low**	- **α**	**Center**	- **α**	**High**
Concentration of astaxanthin (wt %, x_1_)	0.02	0.1	0.2	0.3	0.38
Concentration of emulsifier(wt %, x_2_)	0.2	1	2	3	3.8
Concentration of astaxanthin (wt %,x_3_)	2	10	20	30	38

### Analytical methods

#### Particle-size and polydispersity index (PDI)

Measurement of the mean particle sizes of the nanodispersions and their polydispersity indices was performed at 25°C using a dynamic light scattering particle size analyzer with a measuring range of 0.6–6,000 nm (Malvern series ZEN 1600, Malvern Instruments Ltd., Worcestershire, UK). All measurements were performed on samples (1:10) diluted with deionized water using disposable cuvettes to avoid multiple scattering effects during the measurements. The final particle diameter was calculated from an average of at least three measurements [20].

### Determination of astaxanthin content

#### Sample preparation for astaxanthin determination

To measure the astaxanthin concentrations of the nanodispersions, 0.5 mL of sample was added to 2 mL of a mixture of dichloromethane and methanol (50:50 v/v) in an amber vial with a screw top. The vial was closed tightly and agitated at 400 rpm for 30 min. The mixture was centrifuged at 800 x g for 5 min using a KUBOTA 2010 centrifuge (Tokyo, Japan) at ambient temperature. The extract was decanted. The extraction was repeated two more times [26]. The volume of sample was brought up to 10 mL by the addition of methanol. An aliquot of sample was filtered using a syringe filter (Whatman 25 mm Nylon membrane Syringe Filter, pore size: 1 μm), and 40 μL of filtrate was injected into the HPLC.

#### HPLC analysis

HPLC analysis was performed with an Agilent liquid chromatography system (Agilent Technologies 1200 Series, Waldbroon, Germany), equipped with G13150 Diode Array Detector and a Nova-Pak® C18 ( 3.9 × 300 mm) Waters HPLC column, using an isocratic mobile phase consisting of 85 v/v % methanol, 5 v/v % dichloromethane, 5 v/v % acetonitrile and 5 v/v % water. Detection and quantitative measurement of astaxanthin was performed at 480 nm [20]. The calibration of peak area versus astaxanthin concentration was linear in the range of measured concentrations (R^2^ = 0.9899, n = 5).

#### Calculation of astaxanthin loss

The concentration of astaxanthin in each prepared sample was measured using HPLC after the evaporation process, and the percentage of astaxanthin loss was calculated and considered as a third response variable (Y_3_). The astaxanthin loss (%) was calculated as:

(1) $$\text{Astaxanthin}\;\text{loss}\;\left(\mathrm{wt}\;\%\right)=\left[\left(C*-C\right)/C*\right]\times 100$$

where C is the astaxanthin content of the samples after the evaporation step and C^*^ is the theoretical concentration of astaxanthin, which varied among the prepared samples; it was calculated as:

(2) $$C*=x_{1}/\left(100-x_{3}\right)\;$$

where x_1_ and x_3_ are the corresponding astaxanthin and organic phase concentrations, respectively, for each experiment (Table 1). Thus, in the calculated percentage of astaxanthin loss, all losses that occurred during the conventional and high-pressure homogenization as well as the evaporation steps were considered.

#### Experimental design

Most formulation studies involve the variation of one factor at a time while keeping other factors constant. Such an empirical method is acceptable only when the factors are independent of one another. In factorial designs, all factors can be varied simultaneously; thus, interactions between the variables can be considered through the factorial analysis. The simplest factorial design involves the use of two levels of each variable, allowing only the estimation of linear relationships between independent variables. In situations where quadratic relationships are important, more than two levels should be used in factorial design. However, in these cases, the number of experiments increases considerably. An alternative for these situations is including extra center and star points in a two-level factorial design, known as central composite design (CCD) [4]. Table 4 shows the three emulsion composition variables investigated in this study and their levels. The center point was repeated six times to evaluate the repeatability of the method.

#### Statistical analysis

A response-surface analysis was conducted to study the effect of astaxanthin concentration (x_1_), emulsifier concentration (x_2_) and organic phase concentration (x_3_) on the average particle size (Y_1_), PDI (Y_2_) and astaxanthin loss (Y_3_) of the prepared nanodispersions. The RSM was developed for modeling purposes based on a set of mathematical and statistical methods.

In this work, the independent and dependent variables were fitted to a second-degree polynomial equation (Eq. 3), where Y_i_ is the estimated response, b_0_ is a constant, b_ij_ are the coefficients for each term, and x_i_ are the independent factors.

(3) $$\begin{array}{l} Y_{i}=b_{0}+b_{1}x_{1}+b_{2}x_{2}+b_{3}x_{3}+b_{12}x_{1}x_{2}\\ \;+b_{13}x_{1}x_{3}+b_{23}x_{2}x_{3}+b_{11}x_{1}^{2}+b_{22}x_{2}^{2}\\ \;+b_{33}x_{3}^{2} \end{array}$$

Since it is not possible to predict values when the number of equation variables is greater than that of independent variables, the number of combinations of the independent variables should be higher than 6 , because according to Eq.3, the model contains 10 parameters [27]. Thus, based on the CCD, 20 experiments including 8 cube points, 6 center points and 6 axial points were performed. The distance of the axial points from the central point is known as α. The value of α was calculated by the software and usually depends on the number of independent variables [4,28]. The quality of the fitted models was evaluated by ANOVA, based on the coefficients of determination (R^2^) obtained [4,20]. The corresponding variables were considered more significant if the absolute *F* values were larger and the *p*-values were smaller than *p <* 0.05 [29]. The terms found statistically nonsignificant (*p* > 0.05) were dropped from the initial models, and the experimental data were refitted only to the significant (*p* < 0.05) independent variable effects to obtain the final reduced model. It should be noted that some variables were retained in the reduced model despite their nonsignificance. For example, nonsignificant linear terms were kept in the model if a quadratic or interaction term containing this variable was significant (*p <* 0.05) [10]. Individual and multiple optimization procedures were performed to predict the optimum levels of the independent variables leading to the desired response goals. Verification of models was done by comparing the experimental data with the values predicted by the final reduced models, and the astaxanthin nanodispersions recommended by single and multiple optimization procedures were prepared and evaluated in terms of the studied physicochemical characteristics. All data were treated and analyzed with the aid of the Minitab v.14 statistical package (Minitab Inc., PA, USA).

## Abbreviations

ANOVA: Analysis of variance; C: Astaxanthin content of the samples after the evaporation step; C*: Theoretical concentration of astaxanthin; CCD: Central composite design; HPLC: High pressure liquid chromatography; p: *p*-value (probability value); PDI: Polydispersity index; R: R-squared (coefficient of determination); RSM: Response-surface methodology; Tween 20: Polyoxyethylene sorbitan monolaurate; v/v %: Volume based percentage; wt %: Weight based percentage; x1 x2: x_3,_ Astaxanthin concentration, emulsifier concentration and organic phase concentration, respectively; Y1 Y2: Y_3_, Particle size, PDI and astaxanthin loss of astaxanthin nanodispersions, respectively.

## Competing interests

The authors declare that they have no competing interests.

## Authors’ contributions

NA and CPT have contributed mainly to the experimental design, preparation and characterization of astaxanthin nanodispersion, data interpretation and manuscript preparation of paper. Both authors read and approved the final manuscript.
